# The Emergency Medical Team Operating System — a vision for field hospital data management in following the concepts of predictive, preventive, and personalized medicine

**DOI:** 10.1007/s13167-024-00361-9

**Published:** 2024-04-22

**Authors:** Jan Gaebel, Erik Schreiber, Thomas Neumuth

**Affiliations:** Innovation Center Computer Assisted Surgery (ICCAS), Faculty of Medicine, University Leipzig, Semmelweisstr. 14, 04103 Leipzig, Germany

**Keywords:** Electronic patient record, Emergency medical team, Predictive preventive personalized medicine (PPPM / 3PM), Disaster medicine, Conceptual and technological innovation, Improved individual outcomes, Health policy, Patient needs

## Abstract

In times where sudden-onset disasters (SODs) present challenges to global health systems, the integration of predictive, preventive, and personalized medicine (PPPM / 3PM) into emergency medical responses has manifested as a critical necessity. We introduce a modern electronic patient record system designed specifically for emergency medical teams (EMTs), which will serve as a novel approach in how digital healthcare management can be optimized in crisis situations. This research is based on the principle that advanced information technology (IT) systems are key to transforming humanitarian aid by offering predictive insights, preventive strategies, and personalized care in disaster scenarios. We aim to address the critical gaps in current emergency medical response strategies, particularly in the context of SODs. Building upon a collaborative effort with European emergency medical teams, we have developed a comprehensive and scalable electronic patient record system. It not only enhances patient management during emergencies but also enables predictive analytics to anticipate patient needs, preventive guidelines to reduce the impact of potential health threats, and personalized treatment plans for the individual needs of patients. Furthermore, our study examines the possibilities of adopting PPPM-oriented IT solutions in disaster relief. By integrating predictive models for patient triage, preventive measures to mitigate health risks, and personalized care protocols, potential improvements to patient health or work efficiency could be established. This system was evaluated with clinical experts and shall be used to establish digital solutions and new forms of assistance for humanitarian aid in the future. In conclusion, to really achieve PPPM-related efforts more investment will need to be put into research and development of electronic patient records as the foundation as well as into the clinical processes along all pathways of stakeholders in disaster medicine.

## Introduction

### Background

In the rapidly evolving field of healthcare, the integration of predictive, preventive, and personalized medicine (PPPM/3PM) within emergency and disaster response frameworks has become increasingly critical [1, 2], particularly in the context of sudden-onset disasters (SODs), where the ability to swiftly and effectively deploy humanitarian aid can significantly impact patient outcomes [3–5]. The principles of PPPM as a concept target to provide medical decisions, practices, mechanisms or products tailored to individual patients, or patient groups. Electronic patient record (EPR) systems must support this notion. But not only the patient data should be personalized but the management system itself should include aspects of personalization and individuality, especially in the setting of emergency and disaster medicine, in which medical professionals work under stressful and demanding circumstances.

SODs require a response system that can be deployed quickly and flexibly [6–8]. Over 1 million people have suffered or died from natural disasters since 1995, e.g., earthquakes or floods [5, 9]. Other global perils, like pandemics or widespread illnesses, require actions of PPPM [10]. Most European countries provide special units of humanitarian and disaster aid. These so-called emergency medical teams (EMTs) are “…groups of health professionals (doctors, nurses, paramedics, etc.) treating patients affected by an emergency or disaster.” [11]. They are specialized and trained for specific field hospital operations and are sent to disaster relief operations and support the local healthcare facilities [12].

### Initial activities

In 2017, the project European Modular Field Hospital (EUMFH) aimed to explore how the medical capacity of the Union Civil Protection Mechanism can be improved [13]. Different Member States of the European Union combined their expertise and investigated the possibility to build a common deployable emergency medical team (EMT) level 3. The EMT-working group under the lead of the World Health Organization (WHO) developed standards for EMTs and the variety of services they provide as well as a referral system. This included the goal to create an IT concept for improved data processing and communication. As part of the project, the Innovation Center Computer Assisted Surgery at the University of Leipzig aimed to analyzing and conceptualizing medical and information technology solutions for the collection and forwarding of information for diagnostics, therapy, and hospital management. The project aligns with the WHO’s program for quick, flexible responses to emergencies and aims toward proposing a digital infrastructure. EMT information infrastructure needs to ensure a constant flow of information within the command structure in order to enable the EMT management to make up-to-date decisions on the appropriate use of limited resources in a critical and highly dynamic environment. To solve the challenge, the information technology concepts of existing EMTs of level 1 and 2 or non-European level 3 field hospitals were analyzed. At the time, the first results of the EUMFH project were demonstrated and tested in practice at an EU emergency exercise for medical civil protection modules in 2018. An earthquake with a magnitude of 7.5 in the city of Bucharest was simulated with extensive damage and numerous injuries. The exercise was intended to train the coordination and cooperation of various EMT from different nations. Schreiber et al. recorded, documented, and visualized all patient information within the mobile hospital in digital form, from anamnesis to discharge [14].

### The current initiative

Our current initiative, in relation to the medical capacity of the Union Civil Protection Mechanism, seeks to explore the enhancement through IT solutions that prioritize PPPM principles. The work at hand aims to demonstrate the approach to finding a solution for the described problem by description the current approach to an EPR system for the demanding utilization of emergency medical teams. We illustrate a novel approach to emergency medical team operations, specifically through the lens of an Emergency Medical Team Operating System (EOS). This article contributes to the design and development of advanced health information systems designed to enhance field hospital data management by leveraging PPPM concepts.

## Methods and materials

### System definition and specification

**Fig. 1:**
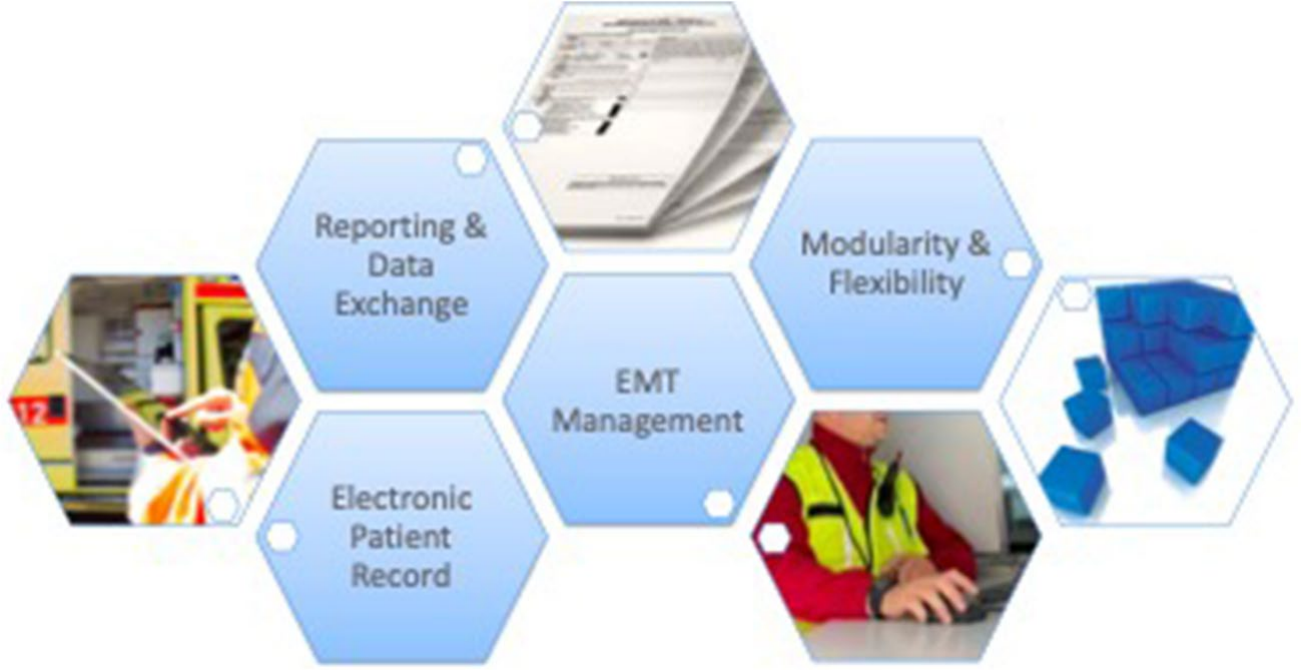
Vision of a comprehensive information system for field hospitals with the four main features that are needed in the field: electronic patient records, emergency medical team (EMT) management, reporting and data exchange, modularity and flexibility

EMTs work under severe constraints and potentially face shortages of every resource imaginable: from medications and personnel to electricity, connectivity, and patient information [15]. Additionally, time pressures make collecting, communicating, and documenting patient data difficult, as does the lack of a standardized framework for the users from many different countries and backgrounds [16]. An electronic patient record must be able to handle all of these challenges while being lightweight, scalable, and highly customizable [17]. Figure 1 illustrates how those requirements, especially features for electronic patient record and EMT management, integrate with data reporting and exchange. Flexibility and modularity in a pre-clinical setting allows for efficient utilization of such a system.

We performed extensive requirement analysis with users from different European EMTs to gather a collective knowledge on the specific needs and aggregated all the feedback to create a common ground for an electronic patient record system. The most important functions to fulfill these requirements are described in the following sections.

### Clinical requirements

An EPR system for EMT must cover all clinical features that normal hospital information systems provide. This ranges from patient admission, treatment planning and documentation to referral documentation and the creation of medical reports. Other, non-medical features should also be included, e.g., material logistics and management, but are not covered within this article. The following paragraphs describe the specific clinical tasks in detail.

**Fig. 2:**
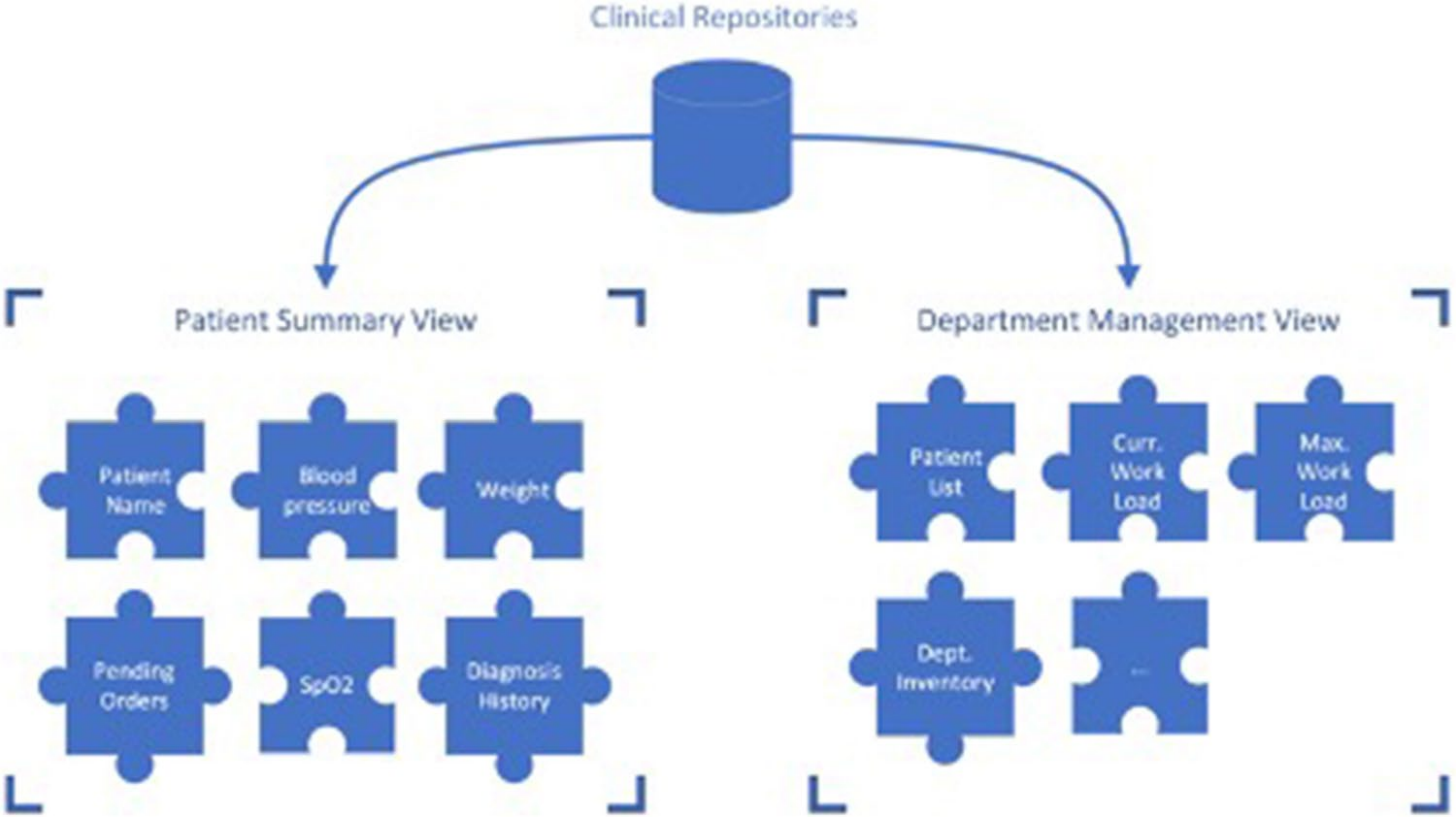
Clinical repositories in Emergency Medical Team Operating Systems (EOS) serve multiple purposes, patients’ master data and clinical data will be applied for administrative or logistical (e.g., stock management) use cases

A) Patient admission and identification: patients treated in an EMT must be admitted to the clinic, and therefore into the EPR. This comprises gathering identifying information and creating unique patient identifiers (PID). For the specific use case in an emergency setting, the triage category of a patient is vital to determine the cases urgency. This data should be available at all time and accessible by different clinical modules of EOS, as illustrated in Fig. 2, where clinical data will be used (or referenced) for other management processes, e.g., stock management or department administration. Thereby, more precise and personalized processes are achievable.
B) Treatment planning and documentation: physicians and nurses attending the patients need to document their assessments and thoughts about a case. They need to be able to write down recommendations and plans for the next measures to take with a specific patient. Also, the performed actions must be stored within an EPR. All diagnostic data from examinations must be available.
C) Referral to different places of therapy and special clinical procedures must be documented and the respective patient information needs to be communicated along all attending staff. This entails the request for diagnostic imaging of surgical treatment by specialized departments of an EMT.
D) The finalization of treatment must be documented and all related information must be provided to the transferring entity (e.g., local hospital, general practitioner, other EMT).

### Regulatory requirements

Data protection is one key aspect that must be implemented by any information system, especially clinical information and electronic patient record systems [18]. A system must provide a way for users demonstrate that the patient has consented to processing of his or her personal data [18].^1^ Processing clinical data must by default only be allowed to personnel directly involved in the treatment. Any other data, e.g., patients from another ward or department, shall by default not be accessible [18].^2^ Any clinical data recorded needs to be consistent and therefore an electronic record system shall maintain a record of processing activities under its responsibility [18].^3^ This includes any changes of individual of clinical data by some user of the system.

EMTs, typically requested by foreign governments and deployed by domestic authorities, must provide regular reports to the respective authority, such as the domestic Ministry of Health or Ministry of Interior, or the local authorities of the country of operation. The “Emergency Medical Team Minimum Data Set Working Group” of the WHO has developed a standardized data sheet by the called EMT Minimum Data Set (MDS) [19, 20]. Reports must adhere to the given format and ideally be automatically generated from existing EPR data.

However, there is a need for commonly agreed standards for what organizational and technical aspects EMTs should adhere to, as well as what features software and hardware should offer, independent of products and manufacturers. Today, there are already globally recognized standards for hardware and information security, such as ISO 27001. Medical data standards are defined internationally by organizations such as HL7, and most vendors comply. The National Electrical Manufacturers Association has published a document on cybersecurity features of networked medical devices, known today as the Manufacturer Disclosure Statement for Medical Device Security.

### Technical requirements

Clinical processes vary among EMTs due to the modular nature of their setup. Therefore, an EPR should also adhere to this structure. Different disaster scenarios involve varying medical conditions and interventions. To ensure prompt and adequate delivery of care with minimal misunderstandings between teams, highly configurable components should be provided. These components can be pre-populated at the onset of a disaster and updated during missions to accommodate changing conditions and requirements. This feature allows for the input of clinical or logistical details to be used in treatment documentation. For example, a list of medications commonly used by EMTs can be included in clinical records after being prescribed to a patient. Additionally, diagnoses established by EMTs (usually a subset of larger classification systems like ICD-10) can be assigned to patients in their electronic records.

The system must be fully functional and have stable performance. As the system is responsible for transmitting critical data to medical personnel, it must be crash-proof. Information must be conveyed reliably and at the same time the transmission and presentation should remain agile. The system must be able to register the right patient, call up their data, and ultimately display the results. In addition, it must respond correctly to requests from the users.

From a usability perspective, it is important to keep the users’ attention on treating the patient and distractions should be avoided. Therefore, the system should be easy to use and ideally intuitive, requiring minimal effort to master. During operation, time and information are critical to a patient’s condition. Accordingly, the system must be able to act and react quickly and appropriately. In order to minimize or avoid latency periods caused by the user’s difficulties in understanding, the display must be clear and easy to understand.

The environment for an EPR inside the infrastructure of an EMT is rather unfavorable. It is usually built outside in mobile housing or tents. Power supply is usually organized by the team itself, by bringing power generators and fuel. However, shortages are always a danger and relying on electronic systems requires a stable power supply, of course. Hence, the application needs to be slim and economical regarding its power demands.

### Non-functional requirements

Qualitative aspects like usability, availability, or robustness are also very important when designing an electronic health record system. They directly affect user acceptance, user performance, and ultimately the patient safety [21]. In general, a system should be designed to be as unrestrictive as possible and as flexible as needed, especially in emergencies.

## Results

### An idea of an optimal system for preclinical use

In this section, we describe the Emergency Medical Team Operating System (EOS) — a specially designed electronic patient record (EPR) system for use during humanitarian aid missions by EMT users. The EOS system is built as a local network in a mobile facility (such as tents or lightweight construction), and users access the core application through client devices (like laptops, tablet computers, or mobile phones). The following paragraphs present an idea of an optimal information system; how it should be set up, how users would interact with it. Subsequently, we present how different restrictions narrow this design. These are the results of customer involvement and extensive requirement analyses over the past years. However, we give an outlook on how a realistic implementation looks like and how it still can produce added value for the patients and clinical users. Those considerations will be reflected in regard to PPPM as well.

Using any tool or assistance system should have minimal impact on clinical users’ work and require minimal effort. The same goes for using an information system. Following the basic principle of medical informatics; the right information should be presented to the right user at the right time in the right format through the right channel. Authenticating oneself and accessing an electronic record in the system should be fairly easy. Stakeholders often expressed the wish to be able to log in easily, only with their ID badge, e.g., using the device camera of a laptop. On the other hand, devices or terminals could always be available for accessing the system giving instant access to the system. Opening the correct record and providing some automatism could be done by proximity sensors attached to the patient bed (e.g., RFID) or simply by scanning patient ID labels. Documentation is automated by speech recognition, data integration from other medical devices (e.g., bedside monitors), or situation awareness.

A seamless integration into clinical workflows is the optimal solution. Data should be gathered automatically, without the need for any user to put in effort to enter specific information into the system. Medical devices that provide data, e.g., a bedside monitor or radiological diagnostics, can be integrated directly and their results can be viewed from the electronic patient record. Principles from modern consumer electronics could provide even more usability and efficiency. Voice control and dictation allow for a very easy way of entering data and providing clinical documentation. Language processing from spoken words could derive further actions and instructions to colleagues.

Mobility and accessibility are major aspects that play an important role in a dynamic setting like an EMT. Clinical users will have to be as flexible as possible in the utilization of medical devices and systems. Access to an electronic patient record system must be possible in different (or changing) situations. The system must therefore be utilized on stationary and mobile devices, presented on small screens or large monitors that present an overview of a complete ward.

In multinational teams working together in a given scenario, supporting systems should be multinational, as well. Not only should the user interfaces support different languages and display their components according to a user’s configuration. Using modern language processing technology, it would be desirable to also have written texts translated, e.g., doctors’ notes or order entries. This would allow not only the multinational staff to have a safer way of communicating but it would also increase compliance by patients since they can understand, too.

### A realistic implementation

A realistic implementation of EOS, however, looks different. Internal and external influencing factors that limit and constrain the features or usability, most of them are restricted by regulatory or organizational aspects. There are also technical constraints. They have less real impact on resulting systems, however.

Electronic patient records must be protected from any malicious access, data leaks, or even manipulation. Therefore, access to the system must be secured with strict methods which contradicts the wish of easy and convenient access. Two-factor authentication would provide a high level of security. This will, however, require access to a second factor (e.g., mobile device) for every employee. The system will have to log off the user after a certain amount of inactivity not to have a terminal or device leaving access to data to unauthorized people.

Deploying the patient record system must be possible on low-level hardware resources that are being shipped with the EMT. A centralized way of providing access to the system, e.g., on a domestic server providing services through the internet, cannot be guaranteed since the operation in a SOD site cannot ensure internet access. Therefore, the operational IT hardware inside the EMT limits the system’s capabilities, as well. EOS is designed as a client–server web application that can be run on the EMTs’ hardware. As a web application, it still can be accessed on different devices and support the need for mobility.

Integration with other systems must be scaled back as well. Since most medical devices restrict their interfaces to linear and direct communication with single clients, a general approach to data integration cannot be embraced. However, EOS will provide interfaces to established standards: for example, HL7 and DICOM [22]. As soon as one medical device allows data exchange in these formats, they can be integrated into the electronic patient record system, as well.

Documentation of clinical activities will be performed in rather conventional ways. Applying text-to-speech technology in a loud or dynamic setting will most likely not present the desired output. However, EOS integrates modern principles of usability and access to allow an easy and efficient way for data entry. This also should result in an increased satisfaction with the system and raise general acceptance of a not optimal but still productive electronic patient record system.

## Impact for predictive, preventive, and personalized medicine

Even when not providing an optimal solution yet, running in a trouble-free environment, establishing an electronic patient record system within EMT structures grants many advantages and added-value. Further reflections to technical advancements or sophistication do not stand in contrast to the mentioned restrictions. However, they must ensure a secured installation and safe application during EMT operation. The following paragraphs provide insight and outlook into the aspects of PPPM in disaster-relief scenarios.

### Toward personalization of EPR

Bringing the advantages of electronic patient records to EMT users demonstrated crucial aspects of system design. The need to provide efficient patient record documentation also produces insight into how EPRs (and their managing systems) can be customized and personalized to fit the users’ needs and wishes. Having a system at hand that allows to adapt to users’ processes and their specific work environment increases the acceptability. Having medical professionals to familiarize with new technology is a complex and demanding challenge. Providing a technology that adapts to the user (at least for certain aspects) simplifies not only the introduction and establishment but in the end, it also increases patient safety.

### Toward predictiveness of emergency patients

Fortunately, sudden-onset disasters occur too rarely to be part of everyday clinical practice. However, this also means that there is less medical knowledge available about SODs. This is further reinforced by poor access to data due to paper-based documentation.

Among other positive effects, the use of an EPR system in an EMT can improve the quality of the documentation and data accessibility and thus helps to accumulate valuable data with each mission that can be analyzed to gain further knowledge how to support personnel, processes, and resource utilization based on clinical evidence. Support can range from direct clinical assistance to simulation of complex cases for training or mission planning [23–25].

Regardless of what is feasible, an ideal EMR for SODs would be capable of tracking the patient journey of each individual patient. This would not only include a very detailed list of complaints, diagnoses, therapies, and aftercare that connects the different steps of the patient journey in a causal way, but also the initial triage status and days between the initial disaster as well as initial events that lead to the patients first admission to the field hospital. During admission, the patient’s information about medication, prior treatments, demography, etc. would be imported digitally from the ambulance and/or the national records via network, mobile app, or similar. Combining this data with the boundary conditions of each mission like type and intensity of the SOD and tracking of treatment success would create an ideal data basis for assessment and prediction. For each patient, all available case data could be compared with those of all previous patients regarding their similarity and used subsequently for assessment of the patients’ current situation, possible therapy options, and prediction of their outcome. This enables various approaches toward individual care and prediction which will be outlined in the following:

#### AI-based triage prioritization

Triage is crucial to utilize the limited EMT resources most efficiently. Patients will be categorized according to their urgency for medical services. Despite that there are strict rules to assign a triage category to a patient, there is no evidence-based evaluation if an assignment was appropriate. By utilizing the aforementioned data basis, an AI-driven tool is able to analyze triage categories [26] and treatment outcome to suggest an optimal triage category for each new patient based on clinical evidence. This approach could help to provide a safer, more resource efficient and more timely treatment.

#### Patient occurrence forecast

From growing patient data (e.g., made available anonymized), clinical informaticians, biometricians, or epidemiologists can learn and provide more insight into the way patient cohorts appear or behave in a humanitarian mission. Prediction of patient emergence is crucial to optimal resource allocation during EMT operation. Having an electronic system available that can predict what is needed in the next hour/day/week on the basis of evidence allows the extremely challenging and demanding work or EMTs and their limited resources to be managed better with a higher chance of all patients receiving the treatment they require.

### Toward prevention of SOD-related reduced treatment quality

Following the AI-based triage prioritization approach, further recommendations can be proposed by EOS countering the lack of resources during SOD and reducing the risk of lowering treatment quality. This might include semi-automated documentation: suggestion of data entries, pre-filling letters or forms based on similar patients with similar results; suggested procedures for diagnosis finding (e.g., to exclude problem A do procedure B); suggest therapy options that were successfully with similar patients.

Keeping track of materials, consumables or medication with an automated documentation process, can also be used to provide an early-warning system for stock shortages. By alerting and proposing delivery for additional materials, danger or shortcomings can be avoided.

These systems and technical features are challenging to implement and set up, however. The aforementioned technical limitations might prevent some of these aspects to be providing altogether. Local infrastructure is likely to be insufficient regarding network availability/quality and data interoperability. For optimal predictions, several missions for different kinds of SOD are required to create a sufficient data basis. It will take some time before the system can reach peak performance.

However, providing decision support in stressful situations can bring several advantages which might surpass the mindset to rely on rather conventional approaches. Clinically speaking, unexperienced or local personnel might be unfamiliar with disaster medicine specifics and can be helped in stressful situations. Limited medical resources are utilized in an optimized way. Patients are presented with a best possible medical care based on clinical evidence. And finally, the underlying system can be trained and updated with each mission’s data to present more and more accurate advice.

## Conclusion and expert recommendation

Our experiences with the concepts and analyses of EOS represent important aspects toward the integration of PPPM principles in emergency and disaster response. This system, designed to provide comprehensive and adaptable support to EMTs in their operations, not only aims to enhance patient care but also provide knowledge about a more resilient, efficient, and tailored technical support to emergency medical services.

Although the development of EOS is still ongoing, our analyses unveiled a lot of potential benefits beyond pure electronic documentation and safe data processing. In the course of our findings, the essential points of implementing PPPM in preclinical emergency and disaster medicine are:

(i) Invest in the establishment of electronic health records and information management in the realm of disaster medicine. A scholarly exchange of stakeholders ranging from medical professionals in the field to executives of health departments or international organizations needs to promote the idea and benefits [27, 28].
(ii) Invest in the development of more sophisticated AI-driven tools for triage and patient management. These tools should be capable of analyzing datasets to predict patient needs and optimize resource allocation in real-time, thereby enhancing the preventive aspect of emergency care [29, 30].
(iii) Invest in regulatory analysis on ownership and how patient data shall be processed inside and outside of European jurisdiction [31].
(iv) Continue to refine the electronic patient records to ensure it is highly customizable and scalable. This will allow for their adaptation to a wide range of emergency scenarios, and the diverse needs of EMTs operating in varied environments and conditions [32].
(v) Work toward integrating of electronic health records with national and international health information systems. This will facilitate an exchange of critical patient information across borders, enhancing the personalized care capacity of EMTs in international disaster response efforts [33].
(vi) Develop comprehensive training programs and simulation exercises for EMTs to familiarize them with the EMT OS. This will ensure that medical teams can fully leverage the system’s capabilities, improving patient outcomes in emergency situations [34].

In conclusion, EOS represents a promising approach in emergency data management, aligning closely with the principles of PPPM. By emphasizing predictive, preventive, and personalized concepts, it demonstrates the potential of digitalization in emergency medicine. However, the realization of its full potential will require concerted efforts in technology development, training, and international cooperation.

## Data/code availability

Not applicable.

## Abbreviations

DICOM: Digital Imaging and Communications in Medicine
EMT: Emergency medical team
EOS: Emergency Medical Team Operating System
EPR: Electronic patient record
EU: European Union
EUMFH: European Modular Field Hospital
FHIR: Fast Health Interoperability Resources
GDPR: General Data Protection Regulation
HL7: Health Level 7
ICD: International Classification of Diseases
ISO: International Standardization Organization
IT: Information technology
MDS: Minimum Data Set
PID: Patient identifier
PPPM/3PM: Predictive preventive personalized medicine
SOD: Sudden-onset disaster
SpO2: Saturation of peripheral oxygen
WHO: World Health Organization

## Footnotes

1 Art. 7 GDPR conditions for consent.

2 Art. 25 GDPR: data protection by design and by default.

3 Art. 30 GDPR: records of processing activities.
